# Gastrointestinal emergencies and monitoring in preterm infants: a review

**DOI:** 10.1080/07853890.2025.2525397

**Published:** 2025-07-01

**Authors:** Yanxia Mao, Yi Yang, Tao Xiong

**Affiliations:** a Department of Pediatrics, West China Second University Hospital, Sichuan University, Chengdu, China; b Key Laboratory of Birth Defects and Related Diseases of Women and Children (Sichuan University), Ministry of Education, Sichuan University,Chengdu, China

**Keywords:** Automated bowel sound, electrogastrography, intra-abdominal pressure, near-infrared spectroscopy, preterm infants

## Abstract

**Background:**

Gastrointestinal emergencies are some of the severe complications in preterm infants, often resulting in fatal patient outcomes due to unintended delays in making precise diagnoses and initiating timely treatment. These delays arise from the non-specificity of observed gastrointestinal events, as well as the shortcomings of the existing monitoring methods used clinically. Therefore, effectively gastrointestinal monitoring should be emphasized to facilitate early detection and prevention of gastrointestinal diseases in preterm infants.

**Methods:**

We collected relevant studies by searching literature published on PubMed, Embase, Cochrane Library, and Google Scholar up to December 2024.

**Results:**

We found that various non-traditional tools have been developed, including automated bowel sound, near-infrared spectroscopy, electrogastrography, intra-abdominal pressure monitoring, fecal microbiome monitoring, fecal biomarkers monitoring, high-frequency heart rate variability. These monitoring methods may provide clues for the early warning of gastrointestinal emergencies, and future research focused on early warnings to improve prognosis of preterm infants is worth conducting.

**Conclusions:**

This review discusses common gastrointestinal events in preterm infants, the current state of gastrointestinal emergencies (necrotizing enterocolitis, digestive tract obstruction or narrowing, and gastrointestinal bleeding), and focuses on the progress and prospects of gastrointestinal monitoring in preterm infants. It could potentially make a positive contribution to preterm infants outcomes and quality of life, particularly in addressing areas where current literature still lacks consensus or detailed analysis.

## KEY MESSAGES

1. The early clinical symptoms of gastrointestinal emergencies often lack specificity. Without timely intervention, these symptoms may escalate, thereby impacting the overall prognosis of premature infants.
2. Various gastrointestinal monitoring tools have been developed, including automatic bowel sounds analysis, near-infrared spectroscopy, gastric electrogastrography, intra-abdominal pressure monitoring, fecal microbiome monitoring, fecal biomarkers monitoring, high-frequency heart rate variability. These monitoring methods may provide clues for the early warning of gastrointestinal emergencies, and future research focused on early warnings to improve prognosis of preterm infants is worth conducting.
3. The development of gastrointestinal monitoring tools will facilitate the growth of subspecialties in gastrointestinal care, ultimately making a positive contribution to neonatal outcomes and quality of life.

## 1. Introduction

Preterm birth account for 10.6% of births worldwide, with extremely preterm infants having a high mortality rate of 21.7% and a necrotizing enterocolitis (NEC) incidence of 8.9% [1]. Preterm infants constituting a distinctive demographic cohort characterized by an underdeveloped gastrointestinal structural and functional framework, exemplify this intricate interplay. The gastrointestinal epithelial mucosal barrier in preterm neonates manifests diminished fortitude, while their complement of gastrointestinal immune cells is marked by both qualitative and quantitative insufficiencies [2]. In the clinical context, these vulnerabilities underscore the susceptibility of preterm infants to gastrointestinal emergencies such as NEC, gastrointestinal obstruction and narrowing and gastrointestinal bleeding. The impact of NEC on affected preterm infants is substantial, with significant consequents following its onset. Mortality rates attributable to NEC range from 10% to 50% [3]. Intestinal atresia is with an incidence of 1 in 5000 and a mortality rate of 5–30% [4]. The occurrence of gastrointestinal bleeding in critically ill neonates is around 10–40%, especially in those with infections, premature birth [5]. These conditions are complex in origin, diverse in clinical presentation, and can significantly impact short-term and long-term prognosis of preterm infants, when diagnosis and treatment are delayed, potentially jeopardizing lives [5,6]. Therefore, gastrointestinal emergencies in preterm infants still require attention. However, early clinical symptoms of gastrointestinal emergencies often lack specificity, potentially escalating without timely intervention and, thereby, impinging upon the overall prognosis of preterm infants. Therefore, it is important to perform gastrointestinal monitoring in preterm infants for early detection of gastrointestinal emergencies.

Traditional gastrointestinal monitoring methods included gastric residual volume, imaging examination, manometry and Multiple intraluminal impedance in combination with pH monitoring (pH-MII) [7–12]. However, these tools are not suitable for the early monitoring of the gastrointestinal emergencies. We found that various novel tools have been developed, including automated bowel sound analysis, near-infrared spectroscopy (NIRS), electrogastrography (EGG), intra-abdominal pressure (IAP) monitoring, fecal microbiome monitoring, fecal biomarkers monitoring, high-frequency heart rate variability (HF-HRV) [13–22]. These monitoring methods may provide clues for the early warning of gastrointestinal emergencies, and future research focused on early warnings to improve prognosis of preterm infants is worth conducting.

This review discusses gastrointestinal events in preterm infants, the current state of gastrointestinal emergencies, and focuses on the progress and prospects of gastrointestinal monitoring.

## 2. Materials and methods

This review aims to discuss common the current state of gastrointestinal emergencies, and focuses on the progress and prospects of gastrointestinal monitoring in preterm infants. A comprehensive literature search was conducted across four major databases: PubMed, Embase, Cochrane Library, and Google Scholar. The search was restricted to articles published up to December 2024. Only studies published in English were included to ensure the accessibility and consistency of the data reviewed. The search strategy employed a combination of specific key terms, including ‘Preterm infants’, ‘Gastrointestinal emergencies’, ‘Automated bowel sound’, ‘Near-infrared spectroscopy’, ‘Electrogastrography’, and ‘Intra-abdominal pressure’. These terms were chosen to capture a broad range of studies focusing on the gastrointestinal emergencies and monitoring in preterm infants.

## 3. Gastrointestinal events

Gastrointestinal manifestations within newborns are quite common. As documented, a substantial 29.1% of neonates encounter manifestations encompassing gastroesophageal reflux or emesis [23]. In neonates, gastroesophageal reflux or emesis can be a common, normal occurrence and not a symptom of intolerance, mainly related to limited gastric capacity, gastric emptying delay, immature muscles, or improper feeding methods [23]. According to data from the USA, there is a 2–26% difference in the prevalence of the diagnosis of gastroesophageal reflux in preterm babies [24]. However, it is important to discern that these events may also be symptomatic of pathological processes, manifesting in clinical scenarios inclusive of but not limited to feeding intolerance, pyloric hypertrophic stenosis, or duodenal atresia. Additionally, it should be noted that clinical events may be rooted in diverse etiological origins. For example, abdominal distension and/or bloody stools may be attributed to a spectrum of conditions spanning from feeding intolerance to protein-induced colitis or, in more severe cases, NEC [25,26]. Differentiating between gastrointestinal events of physiological origin and those of pathological conditions heavily relies on the acquisition of a clinical history and the conduct of a comprehensive physical examination, which is subjective [27]. This reliance on subjective assessment introduces the potential risk of diagnostic inaccuracies, treatment delays, leading to adverse outcomes, or even life-threatening situations [25,28,29]. Therefore, the developments of new gastrointestinal monitoring tools are vital for early detection, diagnosis, and treatment of gastrointestinal diseases in preterm infants, which would be helpful to address this challenge.

## 4. Gastrointestinal emergencies

Gastrointestinal emergencies encompass a spectrum of critical conditions, including but not limited to NEC, digestive tract obstruction or strictures, and gastrointestinal bleeding.

### 4.1. Necrotizing enterocolitis (NEC)

NEC is the most common gastrointestinal emergency in preterm infants [30]. The global incidence of NEC in preterm infants is reported to be around 2–7% [31].

Survivors face a high risk of both short-term and long-term complications, including intestinal stenosis and adhesions, malnutrition, biliary stasis [32]. Effects on the nervous system cannot be ignored, as approximately 25% of NEC survivors experience microcephaly and delayed neurological development [33,34]. A recent study indicated that the complications and effects of NEC persist into childhood, adolescence, and even adulthood [35]. Early enteral nutrition with proper gastrointestinal monitoring has been proposed to prevent NEC, improving the short- and long-term prognosis of preterm infants [36].

### 4.2. Digestive tract obstruction or narrowing

Digestive tract obstruction or narrowing has multiple causes such as: Intestinal atresia, the most frequent cause of neonatal intestinal obstruction worldwide [37]. Hypertrophic pyloric stenosis occurs in 2–4 cases per 1000 live births [38]. The incidence of volvulus in the United States approximates a range of 0.28‰ to 0.35‰ [39]. Recent reported cases indicate that midgut volvulus, a kind of gastrointestinal obstruction or stenosis in preterm infants can be misdiagnosed and lead to poor prognosis [40].

Early identification of the causes and timely surgical intervention can lead to favorable outcome [41–43]. However, due to the lack of objective evaluation for the postoperative enteral nutrition, challenges in feeding and a persistent reliance on parenteral nutrition remain common in neonates. These problems aggravates the postoperative complications[4,44], including NEC, anastomotic leaks, and short bowel syndrome [45,46], as well as the occurrence of intestinal failure-associated liver disease and growth retardation [47]. Thus, the development of objective gastrointestinal monitoring tool to assess enteral nutrition tolerance is very important to reduce relevant complications and improve prognosis.

### 4.3. Gastrointestinal bleeding

The complex causes of gastrointestinal bleeding can include NEC, intestinal malrotation, gastrointestinal vascular malformations, food protein-induced enterocolitis syndrome, congenital coagulation disorders, vitamin K deficiency, sepsis, and others [48]. In the clinical practice, the diagnosis of gastrointestinal bleeding often relies on clinical manifestations symptoms such as hematemesis and melena. It is noteworthy that clinical symptoms usually appear in the advanced stages of the disease, leading to delayed diagnosis and treatment. The delayed diagnosis and treatment contributed to prolonged hospitalization, poor weight gain, and even death [5,6].

In conclusion, traditional monitoring methods based on clinical presentation, signs, and radiology are inadequate for early gastrointestinal function monitoring. Developments of new gastrointestinal monitoring tools are urgent for early diagnosis and timely treatment to improve prognosis in high-risk neonates.

## 5. Progress in gastrointestinal monitoring tools

### 5.1. Traditional gastrointestinal monitoring

#### 5.1.1. Gastric residual volume

The method of monitoring gastric residual volume through gastric tube aspiration is commonly used to assess the feeding tolerance of preterm infants [11]. However, routine measurements may cause harm, such as direct injury to the gastric mucosa, disposal of gastric fluids, medications, and hormones, as well as delayed enteral feeding and prolonged parenteral nutrition [49,50]. Moreover, routine monitoring of gastric residuals has shown mixed results, with some studies indicating limited effectiveness in preventing NEC but potentially increasing feeding interruptions [51,52].

#### 5.1.2. Imaging examination

Plain radiography can be used routinely to evaluate NEC in the neonatal intensive care unit, intestinal obstruction. Nevertheless, it can only provide a snapshot of a single time point, resulting in information on gastrointestinal motility that cannot be effectively assessed. Despite contrast imaging examination can provide functional information on the gastrointestinal tract, it not only increases radiation exposure time but may also leads to contrast agent-related adverse reactions [9].

Abdominal ultrasound enables evaluation of bowel motility, echogenicity, wall thickness, pneumatosis and perfusion, and can detect portal venous gas, ascites, and pneumoperitoneum [10]. It is increasingly employed in diagnosing conditions such as NEC, hypertrophic pyloric stenosis, and intestinal volvulus, as well as for postoperative follow-up of intestinal surgeries [12]. However, the diagnostic accuracy and timeliness of ultrasound can be limited by operator skill, infant’s position, and the relatively time-consuming nature of the procedure [53].

#### 5.1.3. Manometry and multiple intraluminal impedance in combination with pH monitoring (pH-MII)

In the monitoring of gastroesophageal reflux, manometry and pH-MII are the preferred techniques [7,8]. Manometry is primarily employed to evaluate pharyngo-esophageal motility and function. Due to its complexity and requirement for specialized equipment and expertise, it is generally limited to use in specialized centers [7,52]. While manometry is useful in investigating underlying pathophysiological mechanisms of esophageal dysfunction, it does not directly measure or diagnose gastroesophageal reflux [7].

In contrast, pH-MII is increasingly preferred for evaluating gastroesophageal reflux because it comprehensively detects both acid and non-acid reflux episodes. A study found that pH-MII can accurately identify gastroesophageal reflux disease (GERD) in pediatric patients [54]. Clinical decisions guided by pH-MII results have been shown to improve GERD-related symptoms and overall quality of life in infants [55]. Moreover, in neonates, pH-MII possesses prognostic value regarding the duration of GERD symptoms [56]. Thus, pH-MII is valuable for monitoring gastroesophageal reflux, guiding therapeutic interventions, and assessing prognosis in infants. Although GERD and feeding intolerance are distinct entities, pH-MII may offer insights into reflux-associated symptoms among preterm infants, particularly in clinical situations involving gastrointestinal emergencies. Nonetheless, the role of both manometry and pH-MII in evaluating gastrointestinal emergencies in neonates warrants further investigation.

In conclusion, these limitations pose challenges in the timely prevention and diagnosis of gastrointestinal diseases. The development of new gastrointestinal monitoring devices helps address this challenge.

### 5.2. Non-traditional gastrointestinal monitoring

#### 5.2.1. Automated bowel sound analysis

Bowel sounds are a representation of gastrointestinal motility and are considered important signals for monitoring gastrointestinal function. Automated bowel sound monitoring technology has been applied in adult medicine and surgical procedures to monitor postoperative intestinal function recovery, identify early intestinal obstruction, and diagnose diarrhea [57,58], gradually taking shape in neonatal gastrointestinal monitoring [13,14]. In 2022, machine learning methods were used to sample bowel sounds from 68 full-term newborns over 20 h, deriving basic acoustic features for five bowel sound parameters (rate, amplitude, frequency, duration and interval) [14]. An article from 2023 analyzed bowel sounds in 49 preterm or term infants using convolutional neural network models, who on continuous positive pressure ventilation, and identified peristalsis and non-peristalsis sounds [13]. This technology is more objective, sensitive, and capable of continuous monitoring and data sharing compared to doctor auscultation. It may allow preclinical detection of NEC and septicemia [13].

However, there is still a lack of publicly recognized foundational datasets, and improvement is needed in noise reduction and interference elimination [59]. The underlying factors influencing bowel sounds in the preterm infants period remain to be explored [14].

#### 5.2.2. Near-infrared spectroscopy (NIRS)

NIRS is a non-invasive tool and can continuously measure tissue oxygen saturation [60]. NIRS has been found the significance in early detection and diagnosis of NEC. Furthermore, NIRS may play an important role in predicting the survival rates of newborns suffering from NEC. A recent study using NIRS to monitor tissue oxygen saturation for 30 min in 75 preterm infants with gastrointestinal symptoms was conducted [17]. This study found that increased visceral-to-cerebral oxygenation ratio provided a clear diagnosis of NEC. Besides, high variability in local visceral oxygen saturation was significant for early NEC exclusion [17]. An observational study involving 22 preterm infants with surgical NEC revealed that babies who survived after surgery had higher preoperative bowel oxygen levels compared to those who died after surgery. A 10% increase in preoperative bowel oxygen levels was associated with a 4-fold increase in postoperative survival likelihood [15].

However, the role of NIRS in the early discovery of NEC is controversial and has not yet been widely used in clinical practice [26]. Future studies are needed to improve the limitations.

#### 5.2.3. Electrogastrography (EGG)

EGG is a non-invasive technique for assessing gastric myoelectric activity and allows for long-term bedside monitoring. It can assist in evaluating gastrointestinal maturity of preterm infants, thereby reducing the occurrence of feeding intolerance [16,61]. EGG parameters like power spectral density showed potential as biomarkers for assessing preterm infant feeding readiness [16]. In 2022, Chaudhari et al. monitored EGG in 51 preterm infants across three gastric rhythms (bradygastria, normogastria, and tachygastria) and three feeding periods (pre-feeding, during feeding, post-feeding), establishing a relationship between gastrointestinal maturity and neonatal gastric myoelectric activity. EGG can monitor characteristic rhythms associated with the occurrence of gastrointestinal diseases in premature infants. Tachygastria, suggesting gastrointestinal immaturity, related to feeding intolerance and NEC [62]. Two cohort studies indicated that EGG is a repeatable tool for tachygastria in preterm infants [62,63]. Therefore, tachygastria measured by EGG could be as a potential biomarker for pathology for feeding problems in preterm infants.

EGG primarily reflects gastric body and antrum myoelectric activity and is less sensitive to detecting activity in the gastric fundus [64]. Furthermore, there is a lack of unified standards for assessing the accuracy of relevant data and a need to improve the resolution of EGG [65]. Emerging technologies aim to depict gastric motility from the body surface with high spatiotemporal resolution, potentially leading to more objective and accurate monitoring of gastrointestinal function [66].

#### 5.2.4. Intra-abdominal pressure (IAP) monitoring

Elevated IAP exert adverse effects on visceral capillary blood flow [67]. Monitoring IAP serves as a valuable tool for evaluating gastrointestinal function, aiding in early diagnosis of NEC, facilitating timely initiation of enteral nutrition, and expediting postoperative wound recovery in newborn [68,69]. A study involving the monitoring of IAP in 61 preterm infants was conducted. This study reported that a 10% increase in IAP was associated with 85% sensitivity and 63% specificity for diagnosing NEC, thus providing valuable guidance for surgical decision-making [19]. The lower the IAP, the lower the rate of intolerance to enteral nutrition, which helps improve patient outcome [70].

The conventional method of intravesical pressure measurement entails placing a pressure sensor inside the body, which is an invasive procedure that imposes limitations on patient positioning and can be susceptible to interference from the patient’s movements during data collection [71]. In contrast, wireless sensor monitoring devices offer a novel and less intrusive approach. Liao et al. utilized a remote monitoring system with disposable capsule sensors to continuously measure gastric and intestinal pressures in pigs for up to 144 h. This innovative approach allows the monitored subjects to maintain their normal activities during the monitoring process, thus avoiding restrictions [72]. Additionally, Tayebi et al. devised an experiment using transient radar to measure IAP in an external setting [73]. However, both non-invasive monitoring methods remain in the experimental phase. Their accuracy, suitability for human use (especially for preterm infants), and clinical applicability have yet to be fully determined. Further research is required in the future to explore these aspects.

#### 5.2.5. Fecal microbiome monitoring

Fecal examination used to assess the gut health and microbiota of preterm infants. Lower microbial diversity, a decreased abundance of beneficial bacteria, and an increased abundance of pathogenic bacteria are associated with feeding intolerance in preterm infants [21]. However, they are laborious, time-consuming, and only of use for studying culturable microorganisms [74]. The timing of stool sample submission, the use of antibiotics, and the feeding method all influence the test results [75]. These factors limit the specificity and accuracy of the results.

#### 5.2.6. Fecal biomarkers monitoring

The elevation of fecal calprotectin may be related to the migration of neutrophils into the intestinal mucosal lumen, indicating the potential presence of intestinal inflammation [76,77]. A meta-analysis indicated that the sensitivity and specificity of fecal calprotectin for the diagnosis of NEC in preterm infants were 0.91 and 0.93 [22]. However, studies have shown that fecal calprotectin levels in preterm infants exhibit significant variability both between individuals and within the same individual [78]. This variability may be associated with various factors, including the physiological state of the preterm infant, birth weight, feeding methods [79]. In summary, due to the individual differences of fecal calprotectin in preterm infants, its clinical application has certain limitations.

#### 5.2.7. High-frequency heart rate variability (HF-HRV)

Some studies have shown that the natural frequency heart rate variability measured by electrocardiogram can reflect the movement of the upper digestive tract and intestinal immune defense [80]. HF-HRV is associated with the well-functioning neural regulation. Diminished HF-HRV variability is a significant predictor of feeding intolerance or NEC development [20]. This provides another direction for gastrointestinal monitoring. Further studies exploring changes in other vital signs, such as respiration and blood pressure, before the onset of gastrointestinal diseases may be worthwhile. This could help to fully utilize the predictive capabilities of existing monitoring methods for gastrointestinal diseases.

**Figure 1.**
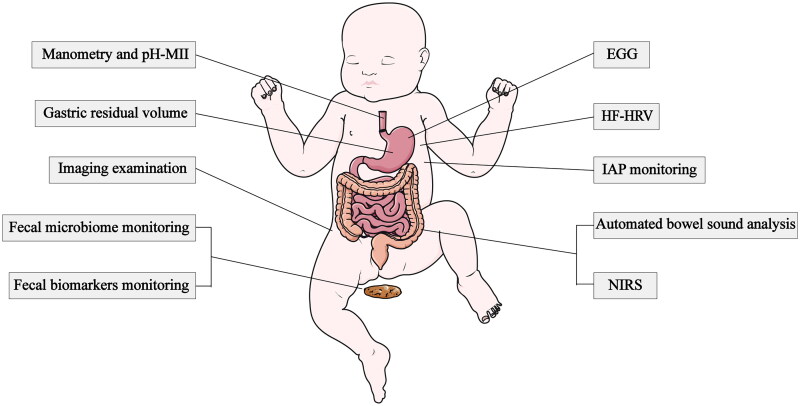
Gastrointestinal monitoring tools. Abbreviations: pH-MII: Multiple intraluminal impedance in combination with pH monitoring, NIRS: near-infrared spectroscopy, EGG: electrogastrography, IAP: intra-abdominal pressure, HF-HRV: high-frequency heart rate variability.

In conclusion, each device has its own advantages and limitations (Table 1; Figure 1). The combined application of two or three of the techniques would overcome their individual limitations and provide quantitative and objective information. A study involving 22 newborns applied a combination of bowel sounds, EGG, and near-infrared spectroscopy [18]. This study indicated that the combined use of these three methods can offer quantitative and objective information about the intestinal developmental process, which suggests potential directions for future research.

**Table 1.** Non-traditional gastrointestinal monitoring tools.

Tool	Author	Years	Study design	Sample size	Object of study	Application	Advantages	Limitations
Automated bowel sound	Zhou [14]	2022	case-control study	68	Term infants	Continuous and real-time monitoring of neonatal gastrointestinal motility	Objective, continuous, Non-invasive and data-sharing	Noise interference, lack of accepted data sets
	Burne [13]	2023	Diagnostic test study	49	Preterm infants and term infants	Allow preclinical detection of NEC and Septicemia		
NIRS	Kuik [15]	2022	Cohort study	22	Preterm infants	Assess the postoperative survival rate after NEC	Non-invasive and convenient	The accuracy and validity of the data are controversial
	van der Heide [17]	2021	Cohort study	75	Preterm infants	Early diagnosis NEC		
EGG	Chaudhari [16]	2022	Cohort study	51	Preterm infants	Assessing Gastrointestinal maturity, related to feeding intolerance, and NEC	Non-invasive, safe, and practical	Low resolution, accuracy need to be verified
IAP	Tanriverdi [19]	2013	Diagnostic test study	61	Preterm infants	Early diagnosis NEC and expediting postoperative wound	Continuous monitoring	Invasive, restricted activities
Fecal microbiome	Hu [21]	2021	Cohort study	97	Preterm infants	The alteration in fecal microbiota and the development of feeding intolerance	Non-invasive, safe	Laborious, time-consuming, for culturable microorganisms
Fecal biomarkers	Qu [22]	2020	Meta analysis	568	Preterm infants and term infants	Fecal calprotectin was used to diagnose NEC	Non-invasive, safe	Clinical application value is Unclear
HF-HRV	Meister [20]	2021	Cohort study	250	Preterm infants	Predict feeding intolerance or NEC development.	Non-invasive, safe	Clinical application value is Unclear
Automated Bowel Sound, EGG, and NIRS	Ortigoza [18]	2018	Cohort study	23	Preterm infants and term infants	Measures indicative of gastrointestinal maturity	Combined three devices and circumvent their individual limitations	None

NEC: necrotizing enterocolitis, NIRS: near-infrared spectroscopy, EGG: electrogastrography, IAP: intra-abdominal pressure, HF-HRV: high-frequency heart rate variability.

## 6. Prospects and challenges of gastrointestinal monitoring

### 6.1. Development prospects

#### 6.1.1. Integration with traditional gastrointestinal monitoring tools

Traditional gastrointestinal monitoring tools remain crucial in clinical practice. For example, the practice of routine gastric residual volume measurement to guide enteral feeding in neonatal wards is widespread [11]. The early monitoring of diseases through the integration of traditional gastrointestinal monitoring tools is gradually advancing. Recent research proposes the integration of sensors into a pig’s gastric tube for *in situ* measurement of gastric fluid and gas, providing an alternative for early disease monitoring, particularly during the acute phase of sepsis [81]. This could have significant implications for the early prevention of NEC.

#### 6.1.2. Mobility and dynamic monitoring

Emerging wearable gastrointestinal monitoring devices greatly expand the scope and applicability of gastrointestinal function monitoring. These devices offer the capability for real-time monitoring in various settings, including home environments, hospitals, and during patient transportation, thereby making substantial contributions to the field of neonatal gastrointestinal care. In 2020, a study used a wearable bowel sound monitoring system to continuously monitor bowel sounds for 24 h in 20 adults. Convolutional neural networks were used to segment bowel sounds, resulting in a dataset that demonstrated high sensitivity [82]. In the future, there is a need to establish a dataset for preterm infants bowel sounds, forming a foundation for dynamic gastrointestinal monitoring.

#### 6.1.3. Safe and non-invasive diagnosis

It is well known that fetal brains can be adversely affected by ionizing radiation, leading to structural and functional defects [83]. Therefore, minimizing radiation exposure during the neonatal period is crucial. Emerging assessment tools for gastrointestinal maturity include automated bowel sound, EGG, NIRS. Moreover, recent research has reported that the oral fluorescent probes can accurate and reliable detection of gastrointestinal disease *in vivo*. It represents a promising non-invasive diagnostic approach [84]. These innovative tools are non-invasive, radiation-free and safe, making them suitable for widespread use in neonatology, offering the potential to improve neonatal outcomes and quality of life.

#### 6.1.4. Integration with metabolomics monitoring

Metabolomics is an emerging and rapidly advancing field within microbiology, offering promising insights for the early detection of diseases such as NEC, lactose intolerance, and gastrointestinal absorption disorders. A review in 2022 mentioned that metabolic exploration in preterm infants can potentially be used to identify reliable biomarkers of NEC both for the early diagnosis and for predicting the risk of adverse clinical outcomes [85]. Besides, studies have shown that detecting hydrogen and methane produced by intestinal microbiota fermenting carbohydrates can be used to detect lactose intolerance in infants [86–88]. The recent development of self-powered, battery-free ingestible biosensing capsules has achieved *in situ*, real-time intestinal glucose monitoring in living subjects [89]. This advancement holds the promise of providing new and effective monitoring tools for the diagnosis of conditions such as malabsorption in the gastrointestinal tract.

#### 6.1.5. Objective monitoring using artificial intelligence or machine learning

With the advent of precision medicine, effective and objective monitoring systems are becoming increasingly possible. A notable example is the use of neural networks to analyze bowel sounds, combining machine learning with gastrointestinal monitoring [13,14,90]. A recent study used smartphones to monitor bowel sounds in 100 healthy adults, making bowel sound monitoring more convenient and intelligent [91]. Furthermore, machine learning models have been employed to identify adult patients at risk of recurrent gastrointestinal bleeding and to predict those most likely to require treatment. These models have demonstrated impressive accuracy rates, with some achieving up to 90% accuracy in their predictions [92]. This approach has the potential for further development in the neonatal population.

### 6.2. Challenges

#### 6.2.1. Further improvement is needed in early-stage research and clinical application of gastrointestinal monitoring devices

Some emerging gastrointestinal monitoring devices are still in the research validation phase and have not been widely applied clinically. For example, bowel sound detection techniques combined with machine learning models have limited research in the preterm infants [57,58]. EGG, IAP still face various limitations. There is no standardized dataset or reference standards available, and the clinical utility remains contentious [86,93,94]. Non-invasive IAP monitoring is still in the research and development phase. Several factors, including potential interference, can lead to false-negative or false-positive results in these monitoring systems. Further refinement and validation are necessary to address these challenges and ensure the reliability and accuracy of non-invasive IAP monitoring methods for clinical use [65,72,95,96]. The future of technology development remains uncertain, leading the potential limitations in the diversity of device selection and the accuracy of final data.

#### 6.2.2. Long-term prognosis remains unknown

Current studies are limited to gastrointestinal monitoring during neonatal hospitalization, leaving a gap in long-term impact research. The long-term prognosis of these infants remains unknown, requiring further emphasis on the long-term effectiveness assessment of neonatal gastrointestinal development.

#### 6.2.3. The gastrointestinal monitoring tools is generally performed in single context

The current gastrointestinal monitoring tools are mainly helpful in the diagnosis of NEC. Thus, the application in other gastrointestinal emergencies has not been further studied, which also provides a certain direction for future development.

## 7. Conclusion

Early gastrointestinal monitoring is crucial for preventing gastrointestinal emergencies. It is of significant importance in reducing neonatal morbidity and mortality. This advancement will promote the development of gastrointestinal sub-specialties, ultimately making positive contributions to neonatal outcomes and quality of life.

## Data Availability

Data sharing is not applicable to this review as the article does not involve the creation or analysis of new data.

## Abbreviations

Ph-MII: Multiple intraluminal impedance in combination with pH monitoring
NEC: Necrotizing enterocolitis
NIRS: Near-infrared spectroscopy
EGG: Electrogastrography
IAP: Intra-abdominal pressure
